# Selection of Antibodies with Tailored Properties by Application of High-Throughput Multiparameter Fluorescence-Activated Cell Sorting of Yeast-Displayed Immune Libraries

**DOI:** 10.1007/s12033-018-0109-0

**Published:** 2018-08-03

**Authors:** Christian Schröter, Jan Beck, Simon Krah, Stefan Zielonka, Achim Doerner, Laura Rhiel, Ralf Günther, Lars Toleikis, Harald Kolmar, Björn Hock, Stefan Becker

**Affiliations:** 1 Antibody Drug Conjugates and Targeted NBE Therapeutics, Merck KGaA, Frankfurter Strasse 250, 64293 Darmstadt, Germany; 2 Institute for Organic Chemistry and Biochemistry, Technische Universität Darmstadt, Alarich-Weiss-Strasse 4, 64287 Darmstadt, Germany; 3 Protein Engineering and Antibody Technologies, Merck KGaA, Frankfurter Strasse 250, 64293 Darmstadt, Germany

**Keywords:** Yeast surface display, Fab display, Affinity, Specificity, Fluorescence-activated cell sorting, Combinatorial immune libraries

## Abstract

In this study, we present a multiparameter screening procedure for the identification of target-specific antibodies with prescribed properties. Based on B cell receptor gene repertoires from transgenic rats, yeast surface display libraries were generated, and high-affinity human antibodies were readily isolated. We demonstrate that specific desirable features, i.e., species’ cross-reactivity and a broad epitope coverage can be integrated into the screening procedure using high-throughput fluorescence-activated cell sorting. We show that the applied screening stringencies translate directly into binding properties of isolated human antibody variants.

**Electronic supplementary material:**

The online version of this article (10.1007/s12033-018-0109-0) contains supplementary material, which is available to authorized users.

## Introduction

Monoclonal antibodies (mAbs) as well as other proteins with antibody-like binding characteristics are among the most successful entities in the biopharmaceutical sector [1]. With more than 45 therapeutic mAbs being marketed, over 50 investigational molecules undergoing late-stage clinical evaluation and forecasted first-marketing approvals of 6–9 mAbs per year, the outstanding clinical efficacy of those molecules is evident [2].

There are several distinct ways for the generation of antibody diversities in order to select for mAb candidates. Sources of antibody gene pools can be synthetic libraries or derived from B cell-receptor (BCR) gene repertoires after immunization requiring either wild-type or transgenic animals [3]. Naïve repertoires are generally derived from peripheral lymphocytes of healthy individuals and can be used for the isolation of antibodies against virtually any given target without the necessity of immunization [4], a beneficial feature that is also evident for synthetic antibody gene pools. However, mAbs identified from such approaches may display low affinities for their cognate antigen, thus requiring further optimization [5–7]. On the contrary, libraries from immunized rodents commonly yield mAbs with high affinity and target specificity [8]. This is due to a strong immune response as a result of repeated challenge with a certain antigen. With the advent of transgenic animals harboring human immunoglobulin loci, fully human antibodies are raised by immunization, obviating the need for time-consuming humanization of mAbs isolated from wild-type animals [9, 10].

Originally, antibodies from immunized rodents were selected using hybridoma technology as introduced by Köhler, and Milstein [11]. B lymphocytes and myeloma cells are fused ex vivo to form immortalized antibody secreting hybridoma cells. Cell culture supernatants can then be screened for antigen-specific candidates. This methodology has been refined over the years and represents a state of the art principle for the isolation of mAbs. Although several clinical candidates were generated by means of this methodology, the discovery process generally suffers from a poor fusion efficiency which results in the loss of the major part of the antibody diversity [12, 13].

A way to overcome this issue is the transfer of the entire diversity into an appropriate in vitro selection system such as phage or yeast surface display (YSD) [14–16]. Phage Display, the most established in vitro selection technology, has already yielded six human antibodies that were granted marketing approval with numerous more being investigated in clinical trials [17]. YSD, another powerful display technology, offers the benefits of a eukaryotic expression host, enabling post translational modifications and the correct folding of complex proteins as well as the compatibility with fluorescence-activated cell sorting (FACS) [18]. Several mAbs against haptens and proteins, such as botulinum toxin, gp120 and ricin were isolated from BCR gene repertoires [18–20], clearly demonstrating the versatility of this approach. Furthermore, yeast mating can be used to shuffle heavy- and light chain diversities thereby yielding novel chain combinations. This potentially results in enhanced affinity of candidate binders [21].

In this study, we established a process for screening antibody repertoires from immunized transgenic rats harboring human variable antibody regions against a cancer-related antigen by YSD. Essentially, a multi parameter high-throughput screening allowed for the selection of a panel of mAbs displaying desired properties that qualifies them as hits for further testing in functional assays.

## Methods

### Immunization of Transgenic Rodents Harboring Human Antibody Variable Regions (OminRats™)

In order to generate antibody repertoires against a human receptor tyrosine kinase (h-RTK), six OmniRats™ (3 male and 3 female, each 11 weeks of age) possessing human IgH and Igκ germline loci [10, 22] were DNA immunized at Aldevron Freiburg (http://www.aldevron.com) using their proprietary vector platform via weekly intradermal injection by gene gun in a 3-week timeframe. In the fourth week, animals were boosted by subcutaneous injection of recombinant h-RTK extracellular domain (OriGene Technologies, details provided in the supplemental data), respectively. At present, we are unable to provide the PDB accession number of the target protein, since it is part of an undisclosed preclinical development project. From a cohort of six (h-RTK) rats, three and six were selected according to high serum titers measured for binding to recombinant target protein by ELISA and sacrificed for collection of secondary lymphoid tissues.

All experimental procedures and animal care were in accordance with EU animal welfare-protection laws and regulations. We confirm that all experimental protocols were approved by a licensing committee from the local government (Landesuntersuchungsamt, Koblenz, Germany).

### Library Generation

All used plasmids, yeast strains and media have been described elsewhere [23, 24]. In brief, modified pYD1 yeast surface display vectors were used (Yeast Display Vector Kit, version D, #V835-01, Thermo Fisher Scientific). Two different vectors for heavy (VH-CH1) and light chain expression (VK/VL-C) were employed for surface expression of Fab-fragments, both under control of a galactose-inducible *GAL1* promoter. A linkage to the yeast cell wall was conducted by a genetic fusion of Aga2p to the *C*-terminus of the CH1 domain, allowing for the covalent surface display of the following N-terminal fusion protein: Xpresstag-(G4S)–VH–CH1-(G4S)–His-(G4S)3-Aga2p. The light chain expression vector encoded an aMFpp8 leader sequence for soluble light chain secretion [25]. In order to generate YSD libraries, gap repair cloning by homologous recombination in two yeast strains was employed [26]. Light chain libraries were harbored in *S. cerevisiae* strain BJ5464 [*MATα URA3-52 trp1 leu2Δ1his3Δ200 pep4::HIS3 prb1Δ1.6R can1 GAL*) (American Type Culture Collection (ATCC; number: 208288)], whereas heavy-chain libraries were harbored in *S. cerevisiae* EBY100 cells [*MATa URA3-52 trp1 leu2Δ1 his3Δ200 pep4::HIS3 prb1Δ1.6R can1 GAL* (*pIU211:URA3*)] (Thermo Fisher Scientific). For library construction, 5 × 10^6^ lymph node cells were used for extraction of total RNA by applying the RNeasy Minikit (Qiagen) according to the manufacturer’s instructions. Isolated total RNA was used as a template for cDNA generation with SuperScript III First-Strand Kit (Thermo Fisher Scientific) with random hexamer oligonucleotides. Amplification of VH and VL sequences was conducted in a nested PCR approach using AccuPrime™ *Taq* DNA Polymerase (ThermoFisher) and 50 µL reaction volume with primers (Table S3) annealing to signal sequences and rat CH1 or human KC/LC regions in the first PCR reaction. Conditions of the first PCR reaction: 94 °C for 120 s, 30 cycles of 94 °C for 30 s, 50 °C for 30 s, and 72 °C for 40 s, and final elongation for 2 min at 72 °C. A second PCR reaction was performed to incorporate 5′ and 3′ overhangs for gap repair cloning (Table S4). To this end, 50 ng of pooled and purified product (Wizard SV Gel and PCR Clean-Up system, Promega) from the first PCR reaction was amplified in the second PCR reaction with degenerated primers annealing to variable heavy- and light-chain regions framework1 and framework4 as it follows: 94 °C for 120 s, 30 cycles of 94 °C for 30 s, 58 °C for 30 s, 72 °C for 40 s and final elongation for 2 min at 72 °C (primer sequences adapted from [17]). After purification (Wizard SV Gel and PCR Clean-Up System (Promega), PCR products and digested YSD plasmids were used for yeast library generation as described by Benatuil et al. [26]. Heavy- and light-chain diversities were introduced into different haploid yeast strains [heavy chain: EBY100 (*MATa*); light chain: BJ5464 (*MAT*α)] with opposite mating types, and in the following combined into diploid cells by yeast mating as described by Weaver-Feldhaus et al. [20]. Library sizes were calculated by plating of serial dilutions using double-selective agar plates.

### Library Screening

Gene expression of transformed yeast cells, grown in SD–Trp–Leu [minimal SD-base (Clontech) supplemented with commercially available dropout mix (Clontech) bearing all essential amino acids except tryptophan or leucine (Clontech), supplemented with 5.4 g/l Na_2_HPO_4_ and 8.56 g/l NaH_2_PO_4_ × H_2_O (EMD Millipore)], was induced by inoculation of SG–Trp–Leu medium and incubation at 20 °C and 200 rpm for 48 h. h-RTK–ECD–His antigen was obtained from OriGene. Additionally, RTK variants were produced using pTT5 mammalian expression vectors and the Expi293 expression system with Expifectamin mediated transient transfection according to the manufacturer´s instructions (Life Technologies). His-tagged m-RTK–ECD fusion protein was purified via metal–chelate affinity chromatography by determining A280 values (HisTrap, GE Healthcare) followed by size exclusion chromatography (SEC) using an ÄKTA Explorer 100 system and a HiLoad Superdex 200 pg 26/60 column (GE Healthcare) with DPBS running buffer yielding high protein purities (≥ 90%) as determined by gel electrophoresis with 12% NuPAGE BisTris gels run in MOPS buffer (Invitrogen) and analytical SE-HPLC (TSKgel SuperSW3000 columns, Merck). Similarly, Fc-tagged proteins (h-RTK-Domain-AB and h-RTK-Domain-BC), were purified using Protein-A chromatography (HiTrap MabSelect SuRe, GE Healthcare). Protein concentrations were determined by UV A280 spectroscopy.

Antigen was either directly labeled with NHS-activated Alexa Fluor 647 or biotinylated (EZ-Link Sulfo-NHS-LC-Biotin; Thermo Fisher Scientific) followed by a second staining step using streptavidin Alexa Fluor 647 conjugate or streptavidin R-phycoerythrin conjugate (Life Technologies). All yeast cell labeling steps were performed for 30 min with 1 × 10^7^ cells per 20 µl on ice. In order to prepare cells for sorting, a washing step with PBS was conducted followed by incubation of cells with antihuman kappa light chain-specific goat F(ab′)2 R-PE [SouthernBiotech, (1:20 diluted)] and either directly labeled or biotinylated antigen at indicated concentrations (Fig. 2).

For the selection of variants exhibiting cross-reactive binding to m-RTK (murine-RTK) and h-RTK (human-RTK), biotinylated h-RTK–ECD–His was applied (RTK, Sort 3) followed by labeling with streptavidin R-phycoerythrin conjugate along with m-RTK–ECD–His Alexa Fluor 647. In a subsequent sort (RTK, Sort 4), repertoires specific to each of the two human subdomains were identified upon coincubation of cells with either biotinylated h-RTK-ECD-Domain-AB-Fc or Alexa Fluor 647-h-RTK-ECD-Domain-BC-Fc, followed by secondary labeling with streptavidin, R-phycoerythrin (SA-PE) conjugate. Cells were washed, resuspended in 500 µl PBS per 1 × 10^7^ cells, and subjected to FACS. Selections were performed using a MoFlo Legacy cell sorter (Beckman Coulter) and Summit 5.3 software.

Sorted cells were re-grown in SD–Trp–Leu and either stored at − 80 °C or utilized for the inoculation of SG–Trp–Leu medium for induction of gene expression and another sorting round. After four rounds of FACS, single cells were plated on SD–Trp–Leu agar plates, and colonies were subjected to sequencing procedure (single colony-sequencing service at Quintara Biosciences) to identify and select abundant clones for subcloning and expression of full-length IgG molecules.

### IgG Production and Purification

Isolated pYD plasmids of selected clones were used for subcloning of VH/VL regions into pTT5 vectors using the Expresso CMV-based system (Lucigen cloning) to allow full-length IgG expression in mammalian cells as described elsewhere [23]. In brief, cloned constructs were expressed in Expi293 cells after Expifectamin-mediated transient transfection according to the manufacturer´s instructions (Life Technologies). After 5 days, supernatants were harvested, and antibodies were purified via Antibody Purification Kit and Spin Columns with Prosep-A Media according to the manufacturers’ instructions (Merck KGaA).

### Cellular Binding

For evaluation of anti-h-RTK antibodies, h-RTK-positive MDA-MB-231 and h-RTK-negative MCF-7 breast cancer cells were seeded into wells (1 × 10^5^ cells/well) and incubated with 15 µg/ml antibody solution in PBS–BSA for 1 h on ice, followed by washing with PBS–BSA. Subsequently, bound antibodies were stained with Alexa Fluor 488-conjugated anti-human Fc-specific detection antibody (Jackson ImmunoResearch, 20 µg/ml in PBS + 1% BSA; incubated for 1 h on ice). Cellular binding of antibodies was finally evaluated by flow cytometry with 5000 counts collected per sample using a Guava EasyCyte HT flow cytometer device (EMD Millipore).

### Biolayer Interferometry (BLI)

Binding kinetics of antibodies against h-RTK–ECD–His were measured at 30 °C and 1000 rpm agitation on the Octet RED System (ForteBio, Pall Life Science). To this end, antibodies (5 µg/ml in PBS) were captured on anti-human Fc (AHC) tips (half-maximal loading; 25 s) followed by sensor rinsing in kinetics buffer that served as background buffer [KB; PBS, 0.1% (v/v) Tween-20 and 1% (w/v) bovine serum albumin (BSA)]. Afterwards, association was measured by incubation of biosensors in varying concentrations of h-RTK–ECD–His-solutions for 400–450 s. Subsequently, dissociation was performed for 1000–1200 s. Analyses of binding kinetics was evaluated by ForteBio data analysis software 8.0 using a 1∶1 binding model after Savitzky–Golay filtering. Similar to kinetic analysis, cross-reactivity profiles for anti-h-RTK antibodies along with antibody isotype control were measured using AHC tips (7.5 µg/ml in PBS, 150 s). After 50 s baseline measurement, association to 100 nM h-RTK–ECD–His and 200 nM m-RTK–ECD–His for 200 s was measured in parallel. High antigen concentrations were chosen for cross-reactivity screening to allow robust qualification antibodies exhibiting decreased binding affinities to m-RTK–ECD–His. After dissociation for 60 s, maximum binding levels were determined. For all experiments, a subtraction control was included so that one biosensor loaded with the respective antibody was associated in KB instead of antigen solution.

### Epitope Binning

The epitope binning ELISA assay was performed to determine whether isolated antibody variants bind to different epitopes and hence compete with another for antigen binding. All incubation steps were carried out with 100 µl sample volume at ambient temperature, or as indicated. For every washing step, wells were washed by applying three times 300 µl 0.05% (v/v) Tween in PBS (PBS-T) using the Biotek ELx405 Select CW. First, flat-bottom polystyrene 96-well plates were coated with antibody solution at 2.5 µg/ml overnight at 4 °C, followed by washing of wells and blocking with 300 µl 1% (w/v) BSA in PBS (PBS-BSA) for 1 h and additional washing. Antibodies and h-RTK–ECD–His were diluted in PBS–BSA (87 and 16 nM, respectively), preincubated for 1 h, and mixtures were added to the antibody-coated wells for 1 h. Wells were washed and incubated with HRP conjugated anti-His6 (Roche, 1:500 in PBS–BSA) for 1 h, followed by additional washing and subsequent addition of TMB Ultra-Step (Thermo scientific) and 2 N sulfuric acid. Optical density was measured at 450 nm using a Paradigm™ plate reader (Beckman Coulter). All antibodies were tested against each other, and negative controls were included where the same antibody was used for both the coating and h-RTK–ECD–His preincubation.

## Results

### Construction of Immune Libraries

**Fig. 1:**
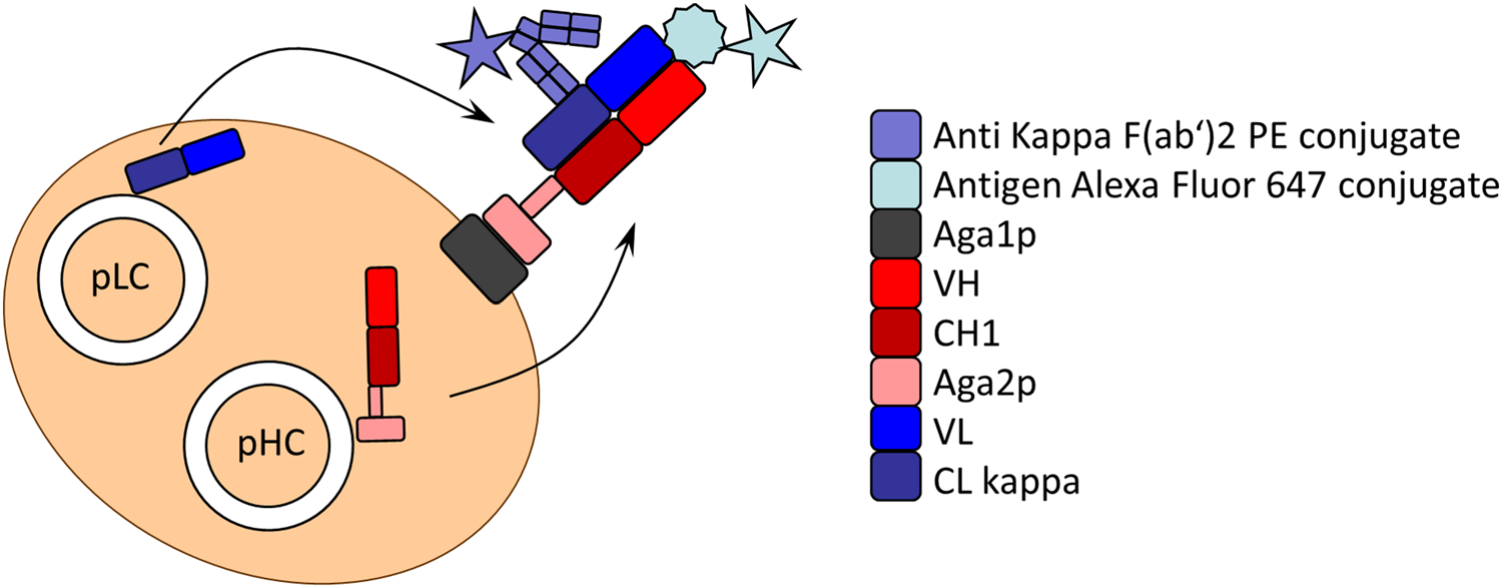
Schematic representation of antibody Fab fragment display on the surface of *Saccharomyces cerevisiae* cells

Besides classical hybridoma technology, the generation of antigen-specific mAbs from immunized animals is also feasible by transferring the animal’s genetic antibody’s diversity into plasmids allowing for in vitro display of antibody fragments, for instance, on phages or yeast cells followed by candidate selection [14–16]. In the current study, YSD libraries were constructed based on antibody repertoires from immunized OmniRats™ [10, 27], which are transgenic for the expression of human antibodies. To this end, animals were immunized with a member of the h-RTK family. After antigen challenge, total RNA was extracted from 2 to 5 × 10^6^ lymph node cells followed by cDNA synthesis with random hexamer primers and VH:VL gene amplification in two consecutive PCR reactions. A second vector construct enables the synthesis and secretion of the light-chain VL–CL domain fusion. Two different yeast strains were used for VH and VL domain library generation, namely EBY 100 (Mat a) for heavy display and BJ5464 cells (Mat α) for light-chain secretion. Upon yeast mating diploid cells are generated where a surface-anchored heavy chain assembles with the secreted light chain to form a Fab fragment (Fig. 1) [23, 28]. Library diversities were calculated to be approximately 2 × 10^9^ unique clones for each of the RTK VH and Vk libraries. Functional Fab molecule-encoding YSD libraries were obtained after fusion of haploid EBY100 and BJ5464 cells by yeast mating, resulting in final library sizes of approximately 3 × 10^8^ unique clones. To monitor display levels of antibody Fab fragments on cells, labeled antibodies directed against the constant region of the displayed light chain were used. Bivariate selections by FACS were performed by incubating cells with Alexa Fluor 647-labeled antigen.

### Selection and Characterization of RTK Targeting Antibodies

**Fig. 2:**
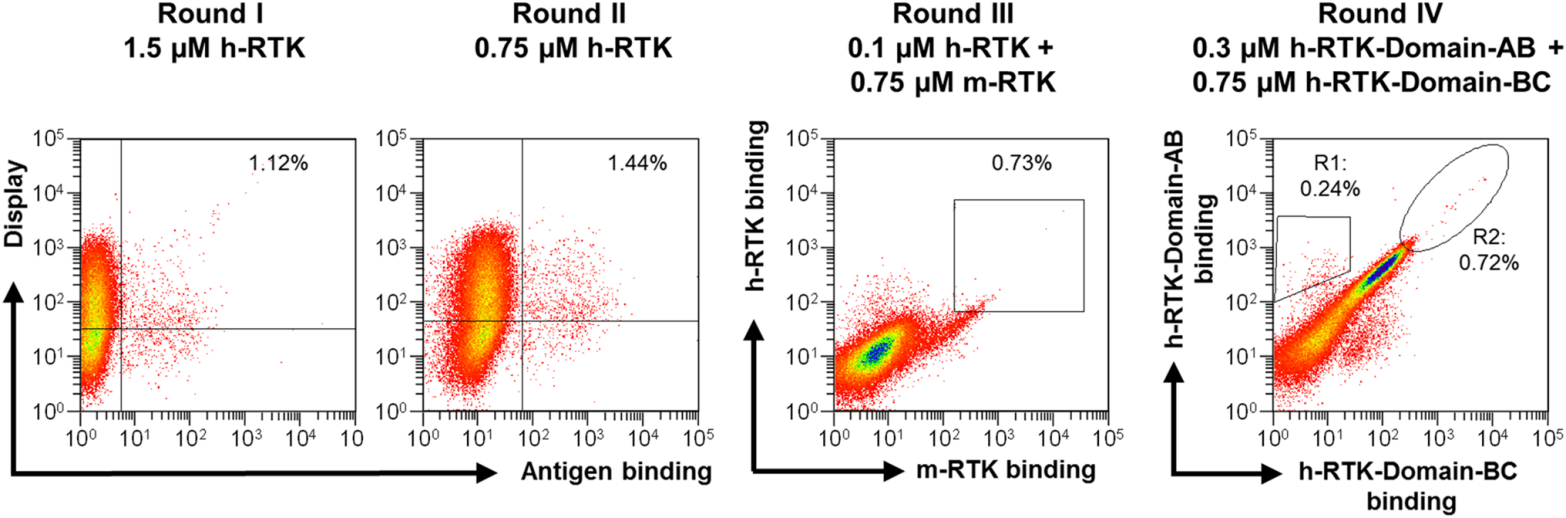
Selection process of libraries generated from lymph node cells of h-RTK-immunized kappa OmniRats™. Bivariate plots of yeast-cells labeled with biotinylated h-RTK–ECD–His, SA-Alexa Fluor 647 (antigen binding) and anti-kappa R-PE for detection of surface expression (display) during round I and II. After round II, cells were double labeled with h-RTK–ECD–His and m-RTK–ECD–His to identify clones exhibiting simultaneous binding to both proteins. After round III, h-RTK subdomain-specific cells were selected by applying two different gates (R1: h-RTK-Domain-A; R2: h-RTK-Domain-B). Yeast cells were coincubated with RTK-Domain-AB (biotinylated) and RTK-Domain-BC (Alexa Fluor 647) subdomains of h-RTK, followed by secondary labeling with SA-PE

For the selection of RTK-targeting antibodies, initial screening was performed using an antigen concentration of 1.5 µM in order to isolate a multitude of different binders and to avoid out-competition of low-affinity binders by high-affinity binders during FACS screening in the first round. During subsequent rounds of sorting, antigen concentrations were reduced to allow for identification and selection of high-affinity binding clones. In these sorts, we aimed at obtaining binders that simultaneously recognize the murine and the human RTK target proteins such that the resulting antibodies can be functionally evaluated in vivo before being considered for a clinical phase I trial. To visualize cross-reactive binding during selections, differently labeled extracellular domains of murine RTK (m-RTK–ECD–His) and extracellular domain of human RTK (h-RTK–ECD–His) were coincubated with cells to identify and select library variants with dual antigen-binding activity (Fig. 2). Stringent gating was applied in round 3 to collect 0.73% of the library cells that were positive for simultaneous m-RTK and h-RTK binding. Since the binding of antibodies to certain epitopes can affect their functional constraints such as antagonism or internalization, it is desirable to evaluate the targeting of distinct epitopes during hit discovery [29].

**Fig. 3:**
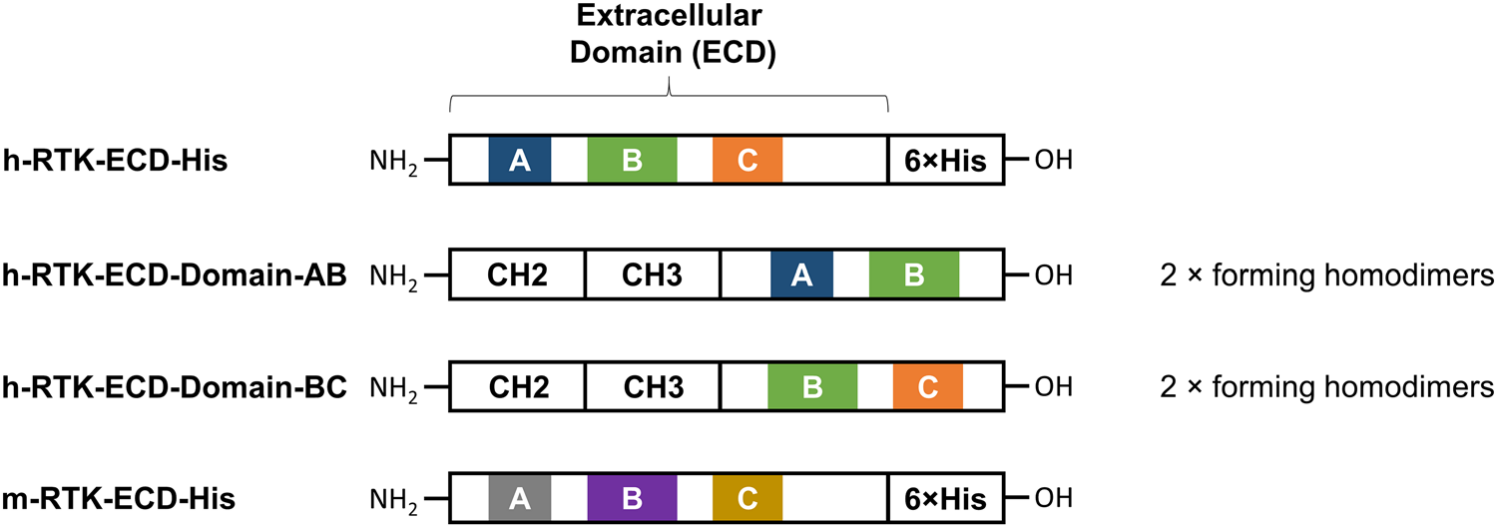
Schematic illustration of recombinantly expressed human- and murine-receptor tyrosine kinase protein (RTK) antigens. Monomeric human (h-RTK–ECD–His) and murine (m-RTK–ECD–His) RTK extracellular domains (ECDs) harbor three subdomains that can be distinguished in domains A (blue), B (green), and C (orange), further separated by four spacer regions, respectively. The ECD is fused on its C-terminus to a hexahistidine-tag for protein purification. Two additional fusion proteins were expressed, so that constant domains CH2–CH3 of human IgG1-Fc were fused to ECD fragments comprising either domains A and B or domains B and C. Both CH2–CH3 fusion proteins assemble into homodimeric molecules connected by two interchain disulfide bonds. (Color figure online)

The h-RTK can be separated into three subdomains A, B and C. To identify subdomain-specific binders, two RTK variants were recombinantly produced that are composed of the RTK-AB lacking the C-domain or of the RTK-BC domain lacking the A domain, respectively (Fig. 3 and Table S1). Hence, a clone that delivers a binding signal for RTK-AB, and no signal for RTK-BC is likely to recognize the A domain. Likewise, clones recognizing both subpopulations are B-domain binders, whereas clones that are stained only with the domain BC likely recognize the C-domain.

**Fig. 4:**
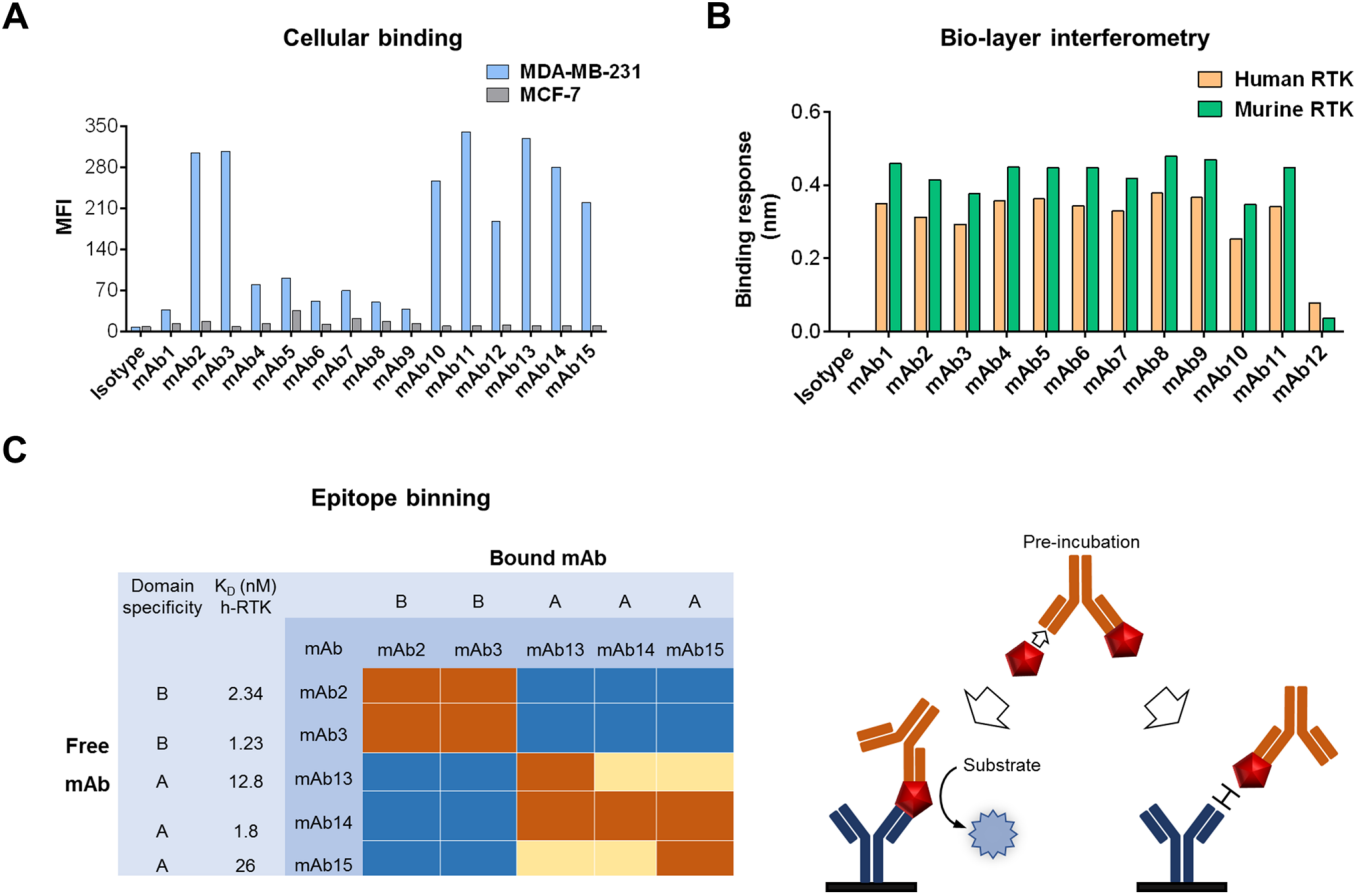
Characterization of selected kappa RTK library variants as full-length antibodies. **a** Cellular binding of selected antibodies was measured by flow cytometry using h-RTK-expressing cancer cell line MDA-MB231 and h-RTK-negative MCF-7 cells. **b** Binding profiles to recombinant h-RTK–ECD–His and m-RTK–ECD–His measured by BLI. **c** Epitope binning analysis of selected antibodies was carried out by ELISA using recombinant h-RTK to determine if antibody variants bind to different epitopes on h-RTK. Antibodies were bound to well surfaces, followed by application of preincubated antibody h-RTK–ECD–His samples (87 and 16 nM, respectively) and detection of bound RTK-ECD-His by anti-His antibody. Binding signals are indicated by colors: blue (high binding, OD_450_ < 0.5), yellow (weak binding, OD_450_ 0.01–0.5), and orange (no binding, OD_450_ > 0.01). Putative subdomain specificities for antibodies derived from the round 4 sorting gates R1 (RTK-Domain-A sort) and R2 (RTK-Domain-B sort) are indicated. Moreover, affinities to hRTK measured by BLI are shown. (Color figure online)

For subdomain discrimination, differentially labeled RTK-AB and RTK-BC were used for cell population staining after the round 3 of sorting. During the round 4, both proteins were applied for real-time identification and simultaneous sorting of subpopulations specific to domains A (gate R1) and B (gate R2) (Fig. 2). In each selection round, about 2 × 10^5^ to 10^6^ cells were isolated. To evaluate sequence diversity after round 4, the heavy- and light-chain sequences of 100 single clones were analyzed as described elsewhere [23] for their CDR diversities. A clustering strategy was applied, where sequences with ≥ 80% identical CDR residues were allocated in the same cluster. Heavy- and light-chain diversities were calculated by dividing the number of clusters by the total number of sequences. Resulting CDR diversities of 8 and 4% (Table S2) indicate sufficient diversity. For further antibody analysis, 15 unique heavy- and light-chain combinations with the highest occurrence were subcloned for soluble expression of full-length IgG1. After expression and purification by protein A chromatography, antibodies were profiled for cellular binding to h-RTK-positive MDA-MB-231 and h-RTK-negative MCF-7 cells (Fig. 4a). All antibodies showed different levels of specific binding to cellular RTK. To analyze the outcome of the library screening strategy that addressed cross-species reactivity, mAbs were subjected to BLI and analyzed for binding to recombinant human and murine RTK proteins (Fig. 4b). Accordingly, the selection strategy was confirmed as almost all antibodies (except for one) showed high-binding signals to both human and murine RTK proteins, while no binding was detected for the isotype control.

To investigate whether isolated antibody variants bind to different epitopes and hence not compete with one another for antigen binding, an ELISA epitope binning assay with five selected antibodies and respective pair combinations was performed exemplarily (Fig. 4c). In principle, when two antibodies recognize different epitopes, increased h-RTK-binding signals are obtained, indicating noncompetitive binding. In accordance with the sorting strategy applied in round 4 (Fig. 2), combining antibodies from RTK-Domain-A-specific and RTK-Domain-B-specific sorts resulted in noncompetitive binding, as indicated by a high binding signal. Consequently, almost no binding signals were measured for antibody pairs from the same subdomain sort. Moreover, binding affinities to full-length h-RTK–ECD–His were determined by BLI, revealing single to double digit nanomolar affinities (Fig. 4c, Fig. S1). Altogether, these findings suggest that yeast-displayed immune-library processing with high-throughput FACS allows efficient multiparameter analysis of library variants, isolation of domain-specific binders and accurate control and regulation of selection conditions and stringencies that translate directly into binding properties of isolated clones.

## Discussion

Since the development of YSD, pioneered by Boder and Wittrup in 1997 [14], a multitude of different proteins were successfully engineered utilizing this versatile platform technology [14, 20, 30–33]. With respect to mAb engineering, YSD proved to be a valid tool for affinity maturation [19, 20, 23] as well as for the direct isolation of high-affinity mAbs based on immune repertoires of rodents [19, 23]. This study demonstrates that antibodies comprising high specificities for their cognate antigen as well as high binding affinities in the nanomolar range can be raised by immunization of transgenic rodents and subsequent library screening using YSD. The main difference between the hybridoma technology and YSD screening lies in the fact that the original pairing of the heavy and the light chain is lost upon separate cloning of the VH- and VL-encoding genes. Hence, the vast majority of YSD clones carries chain pairings that were not evolved during B-cell maturation. Due to loss of information on chain pairing, YSD requires screening of a larger set of clones to identify native VH and VL gene pairs. At this, Wang et al. successfully identified antibodies targeting Ricin using a similar approach [19]. Those authors were able to isolate anti-Ricin antibodies after mouse immunization and yeast surface display selections. They compared the YSD screening outcome with the identification of antigen-specific mAbs from a native antibody repertoire of the draining lymph node and native VH:VL pairings. Interestingly, some isolated variants from YSD recovered native VH:VL pairing, indicating that the heavy- and the light-chain mispairing issues can be overcome by high-throughput screening. Of note, poor biophysical properties of mAbs identified from library approaches are often thought to originate from random VH:VL pairing [34, 35].

In this study, we implemented a multiparameter high-throughput screening strategy, which allowed for the isolation of human mAbs with prescribed properties such as species cross-reactivity as well as defined epitope coverage. By applying a double-staining methodology with differently labeled human and murine RTK, we were successful in selecting variants displaying cross-reactive binding behavior, which was also validated after soluble expression of candidates as full-length IgGs (Figs. 2, 4). Finally, we could verify that antibodies addressing distinct epitopes can easily be obtained by including different subdomains into the sorting strategy. Essentially, this study demonstrates that the sometimes tedious procedure of target-specific mAb characterization can be significantly accelerated since a multitude of essential parameters and desirable antibody features can readily be integrated into the selection process. Furthermore, screening of antibody libraries derived from immunized rodents yields a higher number of antigen-specific antibodies compared to classical hybridoma technology and robust in vitro selection allows for perquisite enrichment of rare antibody specificities [36]. One additional advantage of this strategy is the utilization of human antibody repertoires from transgenic rodents, with no need for sophisticated and time-consuming humanization procedures. Moreover, the methodology described here is also applicable to nontransgenic animals, if needed.

## Conclusions

This study describes a novel screening procedure for the identification of human antibodies from the immunized transgenic animals. We demonstrated that screening for antibodies against diverse epitopes and such show species cross-reactivity can be directly implemented in the screening process. This simplifies the overall identification of hit candidates from antibody repertoires derived from immunization.

## Electronic supplementary material

Below is the link to the electronic supplementary material.

Supplementary material 1 (DOCX 212 KB)

## Data Availability

All data generated or analyzed during this study are included in this published article [and its supplementary information files].

## Abbreviations

AHC: Anti-human FC
BSA: Bovine serum albumin
ECD: Extra cellular domain
FACS: Fluorescence-activated cell sorting
h-RTK: Human receptor tyrosine kinase
HRP: Horseradish peroxidase
mAb: Monoclonal antibody
KC region: Constant kappa light chain region
LC region: Constant lambda light chain region
RTK: Receptor tyrosine kinase
SA-PE: Streptavidin, R-phycoerythrin conjugate
SEC: Size exclusion chromatography
SE-HPLC: Size exclusion high-performance liquid chromatography
YSD: Yeast surface display
PBS: Phosphate-buffered saline
