# Supplementary material for: Selection of Antibodies with Tailored Properties by Application of High-Throughput Multiparameter Fluorescence-Activated Cell Sorting of Yeast-Displayed Immune Libraries
Source: Mol Biotechnol. 2018 Aug 3;60(10):727–35. doi: 10.1007/s12033-018-0109-0 (PMC6132741; doi:10.1007/s12033-018-0109-0)
Supplement: Supplementary file 1 — Supplementary material 1 (DOCX 212 KB) [file 12033_2018_109_MOESM1_ESM.docx]

**Tailoring antibody functions by application of multi-parameter fluorescent-activated cell sorting of yeast-displayed immune libraries**

Christian Schröter^1*^, Jan Beck^2,3*^, Simon Krah^2,3*^, Stefan Zielonka^3*^, Achim Doerner^3^, Laura Rhiel^1^, Ralf Günther^3^, Lars Toleikis^3^, Harald Kolmar^2^, Björn Hock^1^ and Stefan Becker^3+^

^1^Antibody Drug Conjugates and Targeted NBE Therapeutics, Merck KGaA, Frankfurter Strasse 250, D-64293 Darmstadt, Germany^2^

^2^Institute for Organic Chemistry and Biochemistry, Technische Universität Darmstadt, Alarich-Weiss-Strasse 4, D-64287 Darmstadt, Germany

^3^Protein Engineering and Antibody Technologies, Merck KGaA, Frankfurter Strasse 250, D-64293 Darmstadt, Germany

^*^These authors contributed equally to this work

^+^Correspondence to Stefan Becker (Stefan.c.Becker@merckgroup.com)

**Table S1** Recombinant receptor tyrosine kinase (RTK) and interleukin receptor (ILR) protein specifications and sources. Antigen concentrations were determined by photometric measurements on NanoDrop 1000 system by applying molecular weight and extinction coefficient of the respective molecule.

| Name | Specification | Tag | Source | Expression  Host | MW |
| --- | --- | --- | --- | --- | --- |
| h-RTK-ECD-His | Monomeric, human RTK ECD, consisting of subdomains A, B and C | 6×His tag, C-terminally fused | OriGene Technologies, Inc., MD, USA | HEK293 | 45 kDa |
| m-RTK-ECD-His | Monomeric, murine RTK ECD, consisting of domains A, B and C | 6×His tag, C-terminally fused | Internally produced | HEK293 | 43 kDa |
| h-RTK-Domain-AB | Dimeric bivalent, human RTK ECD Fc fusion protein, consisting of domains A and B | human Fc, N-terminally fused | Internally produced | HEK293 | 115 kDa |
| h-RTK-Domain-BC | Dimeric bivalent, human RTK ECD Fc fusion protein, consisting of domains B and C | human Fc, N-terminally fused | Internally produced | HEK293 | 110 kDa |

**Table S2** CDR diversities of analyzed single clones from RTK immune library screening.

|  | **CDR diversity** |
| --- | --- |
| **Heavy Chain** | **7.8%** |
| **Light Chain** | **3.9%** |

CDR diversities determined according to Krah et al [1] by clustering antibody sequences within CDRs to identities of ≥80%. Percentage values were calculated by dividing the resulting number of clusters by the total number of applied sequences.

**Table S3** Oligonucleotide sequences for amplification of antibody variable regions from cDNA.

| Number | Sequence (5′–3′) | |  |
| --- | --- | --- | --- |
| 1 | | ATGGACTGGACCTGGAGGATCC | |
| 2 | | ATGGACTGCACCTGGAGGATCC | |
| 3 | | TCCACGCTCCTGCTGCTGAC | |
| 4 | | GCTACACACTCCTGCTGCTGACC | |
| 5 | | ATGGAGTTTGGGCTGAGCTGG | |
| 6 | | ATGGAATTGGGGCTGAGCTG | |
| 7 | | ATGGAGTTGGGACTGAGCTGGA | |
| 8 | | ATGGAATTTGGCCTGAGCTGG | |
| 9 | | ATGCAGTTTGTGCTGAGCTGG | |
| 10 | | TGAAACACCTGTGGTTCTTCC | |
| 11 | | TGAAGCACCTGTGGTTCTTCC | |
| 12 | | TCATCTTCCTGCCCGTGCTGG | |
| 13 | | GAARTAGCCCTTGACCAGGCATC | |
| 14 | | ATGAGGGTCCCCGCTCAG | |
| 15 | | ATGGAAGCCCCAGCTCAGC | |
| 16 | | GACATGAGAGTCCTCGCTCAGC | |
| 17 | | AAGCCCCAGCGCAGCTTC | |
| 18 | | ATGGTGTTGCAGACCCAGGTC | |
| 19 | | GTCCCAGGTTCACCTCCTCAG | |
| 20 | | CCTGGGAGTTACCCGATTGG | |
| 21 | | CCTCACCCTCCTCACTCAGG | |
| 22 | | CCTCCTCACTCTCTGCACAG | |
| 23 | | TCCTCASYCTCCTCACTCAGG | |
| 24 | | CGTCCTTGCTTACTGCACAG | |
| 25 | | AGCCTCCTTGCTCACTTTACAG | |
| 26 | | CCTCCTCAYTYTCTGCACAG | |
| 27 | | GCTCACTCTCCTCACTCTTTGC | |
| 28 | | CCTCCTCTCTCACTGCACAG | |
| 29 | | GCCACACTCCTGCTCCCACT | |
| 30 | | ATGGCCTGGGTCTCCTTCTAC | |
| 31 | | TGCTCAGGCGTCAGGCTCAG | |

**Table S4** Oligonucleotide sequences for gap repair cloning in yeast and subcloning in mammalian expression vector.

| Number | Sequence (5′–3′) |
| --- | --- |
| 1 | **GCAGGGGATCTGTACGACGATGACGATAAGGGTGGTGGTGGTTCT**GAGGTBCAGCTGGTGCAGTCTGG |
| 2 | **GCAGGGGATCTGTACGACGATGACGATAAGGGTGGTGGTGGTTCT**GARRTSCAGCTGGTRCARTCTGG |
| 3 | **GCAGGGGATCTGTACGACGATGACGATAAGGGTGGTGGTGGTTCT**GAGRTCACCTTGAAGGAGTCTGG |
| 4 | **GCAGGGGATCTGTACGACGATGACGATAAGGGTGGTGGTGGTTCT**GARGTGCAGCTGGTGGAGTCTGG |
| 5 | **GCAGGGGATCTGTACGACGATGACGATAAGGGTGGTGGTGGTTCT**GAGGTGCAGCTGKTGGAGWCYSG |
| 6 | **GCAGGGGATCTGTACGACGATGACGATAAGGGTGGTGGTGGTTCT**GAGGTGCARCTGCAGGAGTCGGG |
| 7 | **GCAGGGGATCTGTACGACGATGACGATAAGGGTGGTGGTGGTTCT**GAGSTGCAGCTRCAGSAGTSSGG |
| 8 | **GCAGGGGATCTGTACGACGATGACGATAAGGGTGGTGGTGGTTCT**GARGTGCAGCTGGTGCAGTCTGG |
| 9 | **GCAGGGGATCTGTACGACGATGACGATAAGGGTGGTGGTGGTTCT**GAGGTACAGCTGCAGCAGTCAGG |
| 10 | **GGAGGAGGGTGCCAGGGGGAAGACCGATGGGCCCTTGGTACTAGC**TGARGAGACRGTGACC |
| 11 | **GCCAGCATTGCTGCTAAAGAAGAAGGGGTAcaaCTCgatAAAAGA**GACATCCAGATGACCCAGTCTCC |
| 12 | **GCCAGCATTGCTGCTAAAGAAGAAGGGGTAcaaCTCgatAAAAGA**GMCATCCRGWTGACCCAGTCTCC |
| 13 | **GCCAGCATTGCTGCTAAAGAAGAAGGGGTAcaaCTCgatAAAAGA**GATRTTGTGATGACYCAGWCTCC |
| 14 | **GCCAGCATTGCTGCTAAAGAAGAAGGGGTAcaaCTCgatAAAAGA**GAAATWGTGWTGACRCAGTCTCC |
| 15 | **GCCAGCATTGCTGCTAAAGAAGAAGGGGTAcaaCTCgatAAAAGA**GACATCGTGATGACCCAGTCTCC |
| 16 | **GCCAGCATTGCTGCTAAAGAAGAAGGGGTAcaaCTCgatAAAAGA**GAAACGACACTCACGCAGTCTCC |
| 17 | **GCCAGCATTGCTGCTAAAGAAGAAGGGGTAcaaCTCgatAAAAGA**GAWRTTGTGMTGACWCAGTCTCC |
| 18 | **ATCAGATGGCGGGAAGATGAAGACAGATGGTGCAGCCACAGTTCG**TTTGATHTCCASYTTGGTCCC |
| 19 | **ATCAGATGGCGGGAAGATGAAGACAGATGGTGCAGCCACAGTTCG**TTTAATCTCCAGTCGTGTCCC |
| 20 | **GCCAGCATTGCTGCTAAAGAAGAAGGGGTACAACTCGATAAAAGA**CAGTCTGTGCTGACTCAGCCACC |
| 21 | **GCCAGCATTGCTGCTAAAGAAGAAGGGGTACAACTCGATAAAAGA**CAGTCTGTGYTGACGCAGCCGCC |
| 22 | **GCCAGCATTGCTGCTAAAGAAGAAGGGGTACAACTCGATAAAAGA**CAGTCTGCCCTGACTCAGCCT |
| 23 | **GCCAGCATTGCTGCTAAAGAAGAAGGGGTACAACTCGATAAAAGA**TCCTATGWGCTGACWCAGCCACC |
| 24 | **GCCAGCATTGCTGCTAAAGAAGAAGGGGTACAACTCGATAAAAGA**TCTTCTGAGCTGACTCAGGACCC |
| 25 | **GCCAGCATTGCTGCTAAAGAAGAAGGGGTACAACTCGATAAAAGA**CTGCCTGTGCTGACTCAGCCC |
| 26 | **GCCAGCATTGCTGCTAAAGAAGAAGGGGTACAACTCGATAAAAGA**CAGCYTGTGCTGACTCAATCRYC |
| 27 | **GCCAGCATTGCTGCTAAAGAAGAAGGGGTACAACTCGATAAAAGA**CAGSCTGTGCTGACTCAGCC |
| 28 | **GCCAGCATTGCTGCTAAAGAAGAAGGGGTACAACTCGATAAAAGA**AATTTTATGCTGACTCAGCCCCA |
| 29 | **GCCAGCATTGCTGCTAAAGAAGAAGGGGTACAACTCGATAAAAGA**CAGRCTGTGGTGACYCAGGAGCC |
| 30 | **GCCAGCATTGCTGCTAAAGAAGAAGGGGTACAACTCGATAAAAGA**CAGSCWGKGCTGACTCAGCCACC |
| 31 | **GGATGGCGGGAACAGAGTGACCGAAGGGGCGGCCTTCGGCTGACC**TAGGACGGTSASCTTG |
| 32 | **GGATGGCGGGAACAGAGTGACCGAAGGGGCGGCCTTCGGCTGACC**TAGGACGGTSASCTTGGTC |

**
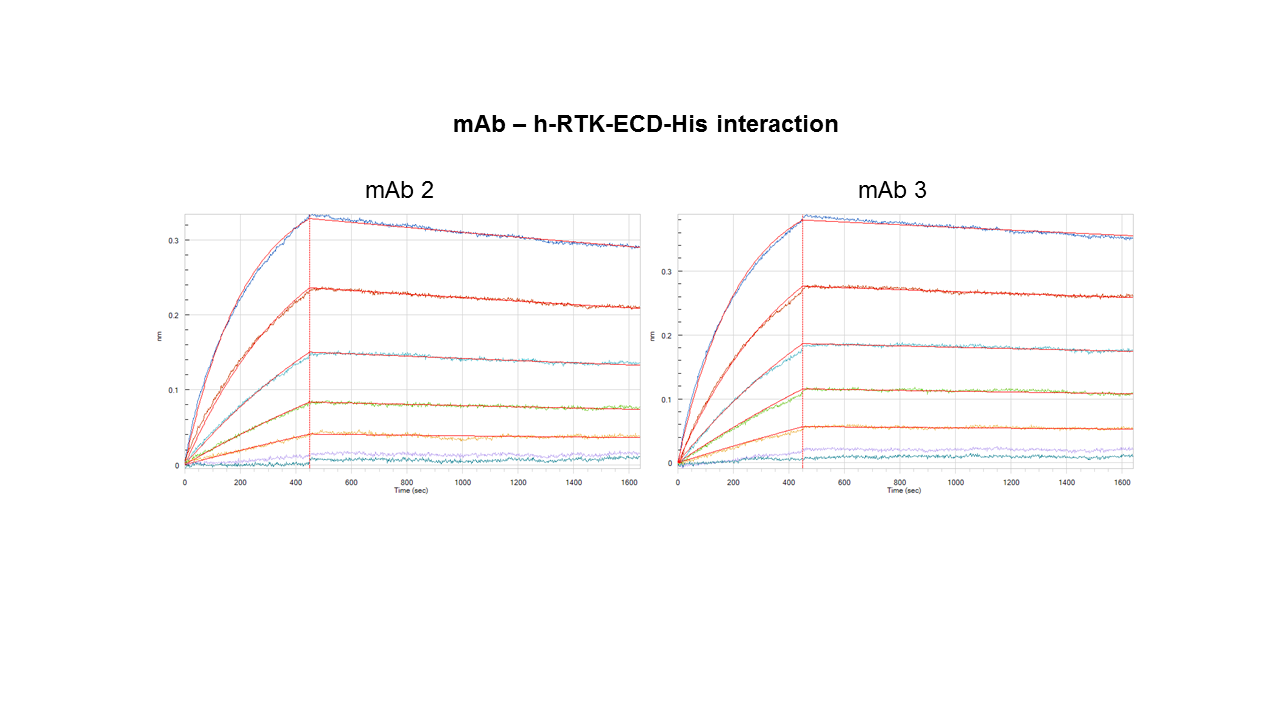
**

**Fig. S1** Exemplary BLI sensorgrams of kinetic analyses of h-RTK-ECD-His binding to immobilized anti-RTK binding antibody variants (mAb 2 and mAb 3). Association with h-RTK-ECD-His at 6.25 nM, 12.5 nM, 25 nM, 50 nM and 100 nM concentrations was measured for 450 s. Dissociation was performed for 1200 s. Binding curves (colored lines) were fitted with a 1:1 interaction model (global fitting, red lines).

References

[1] Krah, S., Schroter, C., Eller, C., Rhiel, L., Rasche, N. *et al.* Generation of human bispecific common light chain antibodies by combining animal immunization and yeast display. Protein Eng Des Sel 2017.
